# Prognostic value of regional myocardial flow reserve derived from ^13^N-ammonia positron emission tomography in patients with suspected coronary artery disease

**DOI:** 10.1007/s00259-021-05459-0

**Published:** 2021-06-30

**Authors:** Elia von Felten, Dominik C. Benz, Georgios Benetos, Jessica Baehler, Dimitri Patriki, Georgios P. Rampidis, Andreas A. Giannopoulos, Adam Bakula, Christoph Gräni, Aju P. Pazhenkottil, Catherine Gebhard, Tobias A. Fuchs, Philipp A. Kaufmann, Ronny R. Buechel

**Affiliations:** Department of Nuclear Medicine, Cardiac Imaging, University Hospital Zurich, Ramistr. 100, CH-8091 Zurich, Switzerland

**Keywords:** Positron emission tomography, Myocardial flow reserve, Myocardial blood flow, Coronary artery disease

## Abstract

**Purpose:**

To assess the prognostic value of regional quantitative myocardial flow measures as assessed by ^13^N-ammonia positron emission tomography (PET) myocardial perfusion imaging (MPI) in patients with suspected coronary artery disease (CAD).

**Methods:**

We retrospectively included 150 consecutive patients with suspected CAD who underwent clinically indicated 13 N-ammonia PET-MPI and who did not undergo revascularization within 90 days of PET-MPI. The presence or absence of a decreased global myocardial flow reserve (i.e., MFR < 2) as well as decreased regional MFR (i.e., ≥ 2 adjacent segments with MFR < 2) was recorded, and patients were classified as having preserved global and regional MFR (MFR group 1), preserved global but decreased regional MFR (MFR group 2), or decreased global and regional MFR (MFR group 3). We obtained follow-up regarding major adverse cardiac events (MACE, i.e., a combined endpoint including all-cause death, non-fatal myocardial infarction, and late revascularization) and all-cause death.

**Results:**

Over a median follow-up of 50 months (IQR 38–103), 30 events occurred in 29 patients. Kaplan–Meier analysis showed significantly reduced event-free and overall survival in MFR groups 2 and 3 compared to MFR group 1 (log-rank: p = 0.015 and p = 0.013). In a multivariable Cox regression analysis, decreased regional MFR was an independent predictor for MACE (adjusted HR 3.44, 95% CI 1.17–10.11, p = 0.024) and all-cause death (adjusted HR 4.72, 95% CI 1.07–20.7, p = 0.04).

**Conclusions:**

A decreased regional MFR as assessed by 13 N-ammonia PET-MPI confers prognostic value by identifying patients at increased risk for future adverse cardiac outcomes and all-cause death.

**Supplementary Information:**

The online version contains supplementary material available at 10.1007/s00259-021-05459-0.

## Introduction

Ischemic heart disease remains the leading cause of death worldwide, and its prevalence is still increasing [1]. Current guidelines recommend non-invasive functional imaging of myocardial ischemia to detect obstructive coronary artery disease (CAD) and guide patient management [2]. Invasive coronary angiography (ICA) or coronary computed tomography angiography (CCTA) offer anatomical information, and with the measurements of fractional flow reserve (FFR) or CT-derived FFR, one can measure pressure gradients and subsequently estimate coronary blood flow [3–6]. By contrast, myocardial perfusion imaging (MPI) using positron emission tomography (PET) allows for accurate quantification of absolute myocardial blood flow (MBF) under rest and stress conditions and calculation of the myocardial flow reserve (MFR) [7].

Several studies have demonstrated the prognostic value of absolute MBF values (hyperemic MBF or MFR) derived from PET-MPI [8–14]. However, the vast majority of these studies have focused on global MBF assessment, encompassing the entire myocardium. While global MBF may be impaired in patients with multi-vessel CAD and those with microcirculatory dysfunction, it may remain largely unaffected in patients at less severe stages of CAD as the focal distribution of coronary artery lesions among the coronary artery tree may lead to only subtle regional differences in MFR.

In the current study, we aim to assess the prognostic value of alterations in regional hyperemic MBF (hMBF) and MFR in patients with suspected CAD.

## Methods

### Study design and population

The present study is a retrospective cohort study comprising consecutive patients from the “Zurich Quantitative PET Registry.” The latter comprises consecutive patients who underwent ^13^N-ammonia PET-MPI at our institution between 2005 and 2015 [14, 15]. We identified all patients who underwent PET-MPI due to suspected CAD and excluded those with incomplete or erroneous PET-MPI datasets and those who underwent revascularization within 90 days after PET-MPI. The local ethics committee approved the study protocol (BASEC-Nr. 2016–00,177), and informed consent for all patients scanned before 2014 was waived. For all patients examined afterward, we obtained written informed consent. If these patients did not want to participate retrospectively, they were excluded from the study.

### PET

As previously described [16], patients underwent ^13^N-ammonia PET-MPI at rest and during adenosine-induced stress at a standard rate (0.14 mg/min/kg) over 7 min with 700–900 MBq of ^13^N-ammonia administered intravenously into a peripheral vein after 3 min into stress. For both rest and stress dynamic (7-min acquisition time with 21 frames, i.e., 9 × 10-s, 6 × 15-s, 3 × 20-s, 2 × 30-s, and 1 × 120-s) and gated datasets (10 min acquisition time divided into eight bins) were acquired in 2D-mode either on a Discovery (LS/RX) or on an Advance PET/CT scanner (both GE Healthcare, Waukesha, WI, USA). Data were reconstructed as static, dynamic, and gated images. Left ventricular ejection fraction (LVEF) was calculated from the gated datasets.

### Data analysis

Images were transferred to a dedicated workstation for analysis (Advantage Workstation, Version 4.5, GE Healthcare) and analyzed regarding the presence or absence of localized fixed and/or reversible regional perfusion defects on the semiquantitative images (i.e., semiquantitative scar and/or ischemia) [17, 18]. Quantitative blood flow analysis has been previously described [16]. In brief, we used PMOD (Version 3.7; PMOD Technologies Ltd., Zurich, Switzerland) to calculate from the dynamic datasets rest and hyperemic MBF (hMBF) for each myocardial segment based on a 17-segment model, applying a two-compartment model [19] corrected for spill-over and partial volume effects. MFR was calculated as the ratio of hyperemic over rest MBF. The latter was corrected for the rate pressure product. Of note, referring and treating physicians were informed of the presence and extent of semiquantitative ischemia and scar, hMBF, global MFR, and LVEF as part of routine clinical reporting.

We defined a decreased global MFR as MFR < 2 [7, 8] and a decreased regional MFR as ≥ 2 adjacent segments with an MFR < 2. Similarly, we defined a decreased global hMBF as MBF < 2 ml/min/g [7] and a decreased regional hMBF as ≥ 2 adjacent segments with hMBF < 2 ml/min/g.

On this basis, we defined the following groups: “MFR group 1” comprises patients with global and regional MFR ≥ 2, “MFR group 2” those with global MFR ≥ 2 but regional MFR < 2, and “MFR group 3” includes patients with global MFR < 2 and regional MFR < 2. Similarly, “hMBF group 1” includes patients with global and regional hMBF ≥ 2 ml/min/g, “hMBF group 2” those with hMBF ≥ 2 but regional hMBF < 2 ml/min/g, and “hMBF group 3” those patients with global hMBF < 2 ml/min/g and regional hMBF < 2 ml/min/g.

### Follow-up

Follow-up data were obtained via telephone interviews with the treating physicians and via the in-house clinical information system. The primary endpoint (i.e., major adverse cardiac events [MACE]) was a composite of all-cause death, non-fatal myocardial infarction, and late revascularization (i.e., > 90 days after PET-MPI). The secondary endpoint was all-cause death.

### Statistical analysis

Continuous data are expressed as mean ± standard deviation (SD) or median and interquartile range (IQR) if not normally distributed. The two-sided t-test was used to compare normally distributed continuous data and the Mann–Whitney-U test for non-parametric continuous data. The chi-squared test was used to analyze the distribution of categorical variables. Pre-test probability for CAD was calculated retrospectively according to the European Society of Cardiology [2]. Differences in survival over time were analyzed using the Kaplan–Meier method, with the log-rank test applied to compare the survival curves. Univariable Cox proportional hazard regression models were used to assess the impact of variables on clinical endpoints. Additionally, backward conditional multivariable Cox regression analysis was applied to identify independent predictors. Significant predictor variables from the univariable analysis were included in the first model. In each subsequent model, the nonsignificant variable with the highest p-value was excluded leaving in the final model only predictor variables with a p-value < 0.1 (Supplementary Table 1). The regression results are presented as hazard ratios (HRs) and their 95% confidence intervals (CI). Variables that were not available for all patients (i.e., LVEF) were not included in the multivariable analysis. The Kendall-Tau test was used to test for correlation among quantitative PET metrics. Variation inflation factors (VIF) were calculated for quantitative PET metrics to test for relevant multicollinearity, and a VIF < 10 was considered tolerable [20]. The *Benjamini*–*Hochberg* procedure was used to control the false discovery rate (FDR) [21]. First, all p-values of the tests are ordered ascendingly and given a rank i. Critical values are then calculated as *(i/m)q*, where *i* is the rank of the test, *m* is the total number of tests, and *q* is the level on which the FDR is controlled. In the present study, the FDR was controlled at the level *q* = 0.1. The test’s rank with the highest p-value equal to or lower than its critical value (i/m)q is defined as *k*. For all tests ranked ≤ *k*, the null hypothesis is then rejected. SPSS software (version 25, IBM Corporation, Armonk, NY) was used for all statistical analysis.

## Results

### Study population

**Fig. 1:**
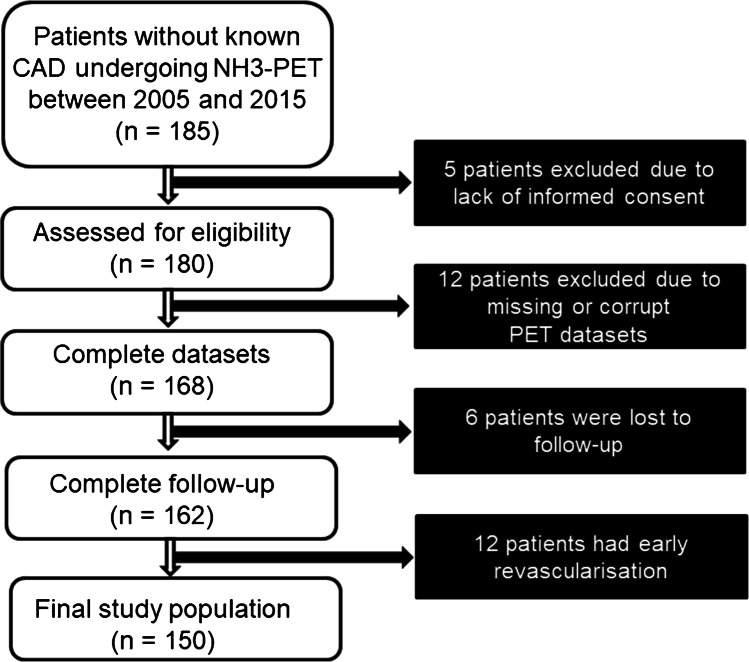
CONSORT diagram of patient enrollment

A total of 185 patients who underwent ^13^N-ammonia PET-MPI due to suspected CAD were identified from the registry. Of these, 5 (2.7%) patients did not provide written informed consent, 12 (6.5%) patients were excluded because of missing or corrupt PET datasets, and 6 (3.2%) patients were lost to follow-up. Additionally, 12 (6.5%) patients who underwent revascularization within 90 days after the PET-MPI examination were excluded. Thus, 150 patients were included in the final analysis (Fig. 1).

The baseline characteristics of the study population are given in Table 1.

**Table 1:** Patient baseline characteristics

	All patients (n = 150)	Patients without MACE (n = 121)	Patients with MACE (n = 29)	p-value	(i/m)q
Age (years)	64 ± 11	62 ± 10	68 ± 12	0.009	0.005
Male sex	82 (54.7)	61 (50.4)	21 (72.4)	0.033	0.014
Body mass index (kg/m^2^)	28 ± 6.6	27.8 ± 6.9	28.9 ± 5.2	0.453	0.068
Pre-test probability for CAD	16% (IQR 11–27)	16% (IQR 10–27)	25% (IQR 14–32)	0.024	0.009
Symptoms					
Typical angina	39 (26)	31 (25.6)	8 (27.6)	0.118	0.018
Atypical angina	14 (9.3)	11 (9.1)	3 (10.3)		
Non-anginal chest pain	41 (27.3)	38 (31.4)	3 (10.3)		
No chest pain	56 (37.3)	41 (33.9)	15 (51.7)		
Dyspnea	50 (33.3)	40 ( 33.1)	10 (34.5)	0.884	0.100
Palpitations	17 (11.3)	15 (12.4)	2 (6.9)	0.401	0.055
Fatigue	8 (5.3)	7 (5.8)	1 (3.4)	0.615	0.077
Syncope or presyncope	15 (10)	11 (9.1)	4 (13.8)	0.448	0.064
Risk factors					
Hypertension	96 (64)	74 (61.2)	22 (75.9)	0.138	0.023
Dyslipidemia	64 (42.7)	54 (44.6)	10 (34.5)	0.321	0.041
Diabetes	24 (16)	17 (14)	7 (24.1)	0.183	0.032
Positive family history	41 (27.3)	35 (28.9)	6 (20.7)	0.371	0.050
Smoking	49 (32.7)	40 (33.1)	9 (30.1)	0.835	0.095
Cardiac medication					
Antithrombotics	59 (39.9)	47 (38.8)	12 (41.4)	0.802	0.091
Anticoagulants	27 (18)	20 (16.5)	7 (24.5)	0.338	0.045
Betablockers	68 (45.3)	53 (43.8)	15 (51.7)	0.441	0.059
Calcium antagonists	29 (19.3)	22 (18.2)	7 (24.1)	0.466	0.073
ACE inhibitors	60 (40)	46 (38)	14 (48.3)	0.311	0.036
Lipid-lowering drugs	57 (38)	45 (37.2)	12 (41.4)	0.676	0.082
Nitrates	7 (4.7)	6 (5)	1 (3.4)	0.729	0.086
Diuretics	36 (24)	26 (21.5)	10 (35.5)	0.141	0.027

Values given are mean, median or absolute numbers with standard deviations, interquartile ranges (in brackets) or percentages (in brackets), respectively. No variables remained significant at an FDR-controlled level q of 0.10. *ACE =* angiotensin-converting-enzyme

### Imaging findings

Imaging findings stratified by the pre-defined MFR and hMBF groups are presented in Table 2. Global and regional MFR and global and regional hMBF differed significantly between the MFR and the hMBF groups, while semiquantitative findings did not. Of note, there were no patients with preserved regional but decreased global MFR. LVEF calculation from PET data was feasible in 125 (83.3%) patients, and LVEF differed significantly among the hMBF but not the MFR groups. Of the 25 patients with missing LVEF values, 11 were classified in MFR group 1, 5 in MFR group 2, and 9 in MFR group 3, and 6 in hMBF group 1, 11 in hMBF group 2, and 8 in hMBF group 3.

**Table 2:** Findings from PET-MPI stratified by groups (n = 150)

Finding	All patients (n = 150)	MFR group 1 (n = 52)	MFR group 2 (n = 49)	MFR GROUP 3 (n = 49)	p-value	(i/m)q	hMBF group 1 (n = 34)	hMBF group 2 (n = 48)	hMBF group 3 (n = 68)	p-value	(i/m)q
Semiquantitative ischemia	20 (13.3)	6 (11.5)	6 (12.2)	8 (16.3)	0.575	0.1	3 (8.8)	5 (10.4)	12 (17.6)	0.359	0.1
Semiquantitative scar	20 (13.3)	6 (11.5)	5 (10.2)	9 (18.4)	0.296	0.089	1 (2.9)	7 (14.6)	12 (17.6)	0.114	0.089
**Global MFR**	**2.4** **(1.7–2.8)**	**3.3** **(2.7–3.5)**	**2.3** **(2.1–2.5)**	**1.5 (1.1–1.7)**	** < 0.001**	0.011	**3** **(2.5–3.3)**	**2.7** **(2.1–3.2)**	**1.9** **(1.4–2.3)**	** < 0.001**	0.011
**Global MFR < 2**	**49 (32.7)**	**0 (0)**	**0 (0)**	**49 (100)**	** < 0.001**	0.011	**4 (11.8)**	**9 (18.8)**	**36 (52.9)**	** < 0.001**	0.011
**Global hMBF (ml/min/g)**	**2.1** **(1.6–2.6)**	**2.6** **(2.3–3)**	**2** **(1.6–2.3)**	**1.7** **(1.2–2)**	** < 0.001**	0.011	**3** **(2.7–3.3)**	**2.4** **(2.2–2.5)**	**1.5** **(1.3–1.9)**	** < 0.001**	0.011
**Global hMBF < 2 ml/min/g**	**68 (45.3)**	**7 (13.5)**	**25 (51)**	**36 (73.5)**	** < 0.001**	0.011	**0 (0)**	**0 (0)**	**68 (100)**	** < 0.001**	0.011
**Regional MFR < 2**	**98 (65.3)**	**0 (0)**	**49 (100)**	**49 (100)**	** < 0.001**	0.011	**11 (32.4)**	**26 (54.2)**	**61 (89.7)**	** < 0.001**	0.011
**Regional hMBF < 2 ml/min/g**	**116 (77.3)**	**29 (55.8)**	**42 (85.7)**	**45 (91.8)**	** < 0.001**	0.011	**0 (0)**	**48 (100)**	**68 (100)**	** < 0.001**	0.011
**LVEF (%)**	51 (46–60)	53 (49–59)	52 (46–62)	49 (42–58)	0.107	0.078	**58** **(55–62)**	**53** **(48–60)**	**48** **(41–58)**	** < 0.001**	0.011

Values given are absolute numbers and percentages (in brackets) or median and IQR (in brackets). Variables significant at an FDR-controlled level q of 0.10 are highlighted in bold

### Outcome

The median follow-up time was 50 months (IQR 38–103). During the follow-up period, 30 MACE occurred in 29 (19.3%) patients: 5 patients (3.3%) underwent late revascularization, 4 (2.6%) patients suffered non-fatal myocardial infarction, and 21 (14%) patients died. One patient experienced two MACE (late revascularization and death). Incidence rates according to the MFR and hMBF groups are provided in the Supplementary Table 2.

**Fig. 2:**
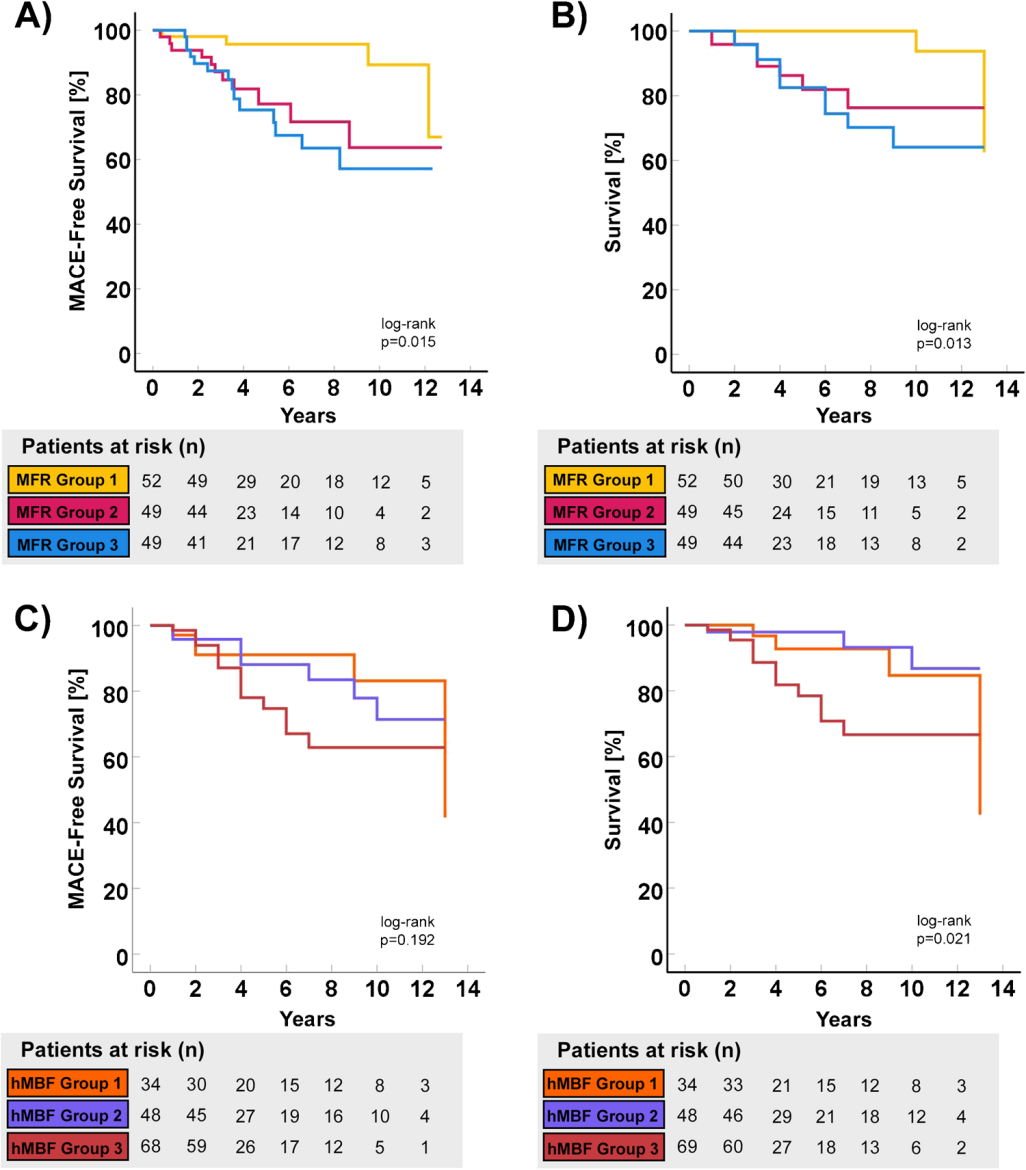
Kaplan-Meyer curves of MACE-free survival stratified by regional and global myocardial flow reserve (MFR) (**A**) and hyperemic myocardial blood flow (hMBF) (**C**) and overall survival stratified by regional and global MFR (**B**) and hMBF (**D**) MFR Group 1: global and regional MFR ≥ 2; MFR Group 2: global MFR ≥ 2 and regional MFR < 2; MFR Group 3 global and regional MFR < 2. hMBF Group 1: global and regional hyperemic MBF ≥ 2 ml/min/g; hMBF Group 2 global hyperemic MBF ≥ 2 ml/min/g and regional hMBF < 2 ml/min/g; hMBF Group 3 global and regional hMBF < 2 ml/min/g

The log-rank test revealed significant differences in event-free and overall survival (p = 0.015 and p = 0.013, respectively) across the various MFR groups (Fig. 2). Specifically, MFR group 1 differed significantly from MFR group 2 and MFR group 3 regarding event-free survival (p = 0.013, (i/m)q = 0.067 and p = 0.003, (i/m)q = 0.033, respectively) and overall survival (p = 0.016, (i/m)q = 0.067 and p = 0.002, (i/m)q = 0.033, respectively). By contrast, no significant difference in event-free or overall survival was found between MFR group 2 and MFR group 3 (p = 0.606, (i/m)q = 0.1 and p = 0.566, (i/m)q = 0.1, respectively).

For the various hMBF groups, the log-rank test revealed significant differences for overall survival (p = 0.021) but not for event-free survival (p = 0.192). Event-free survival did not differ significantly between the different hMBF groups (p = 0.861, (i/m)q = 0.1 for hMBF group 1 versus 2, p = 0.16, (i/m)q = 0.067 for hMBF group 1 versus 3, and p = 0.146, (i/m)q = 0.033 for hMBF group 2 versus 3). Overall survival in hMBF group 2 differed significantly from hMBF group 3 (p = 0.01, (i/m)q = 0.033), while differences in overall survival between hMBF group 1 and 2 (p = 0.368, (i/m)q = 0.1) as well as between hMBF group 1 and 3 (p = 0.133, (i/m)q = 0.067) were not significantly different.

Results of the cox-regression analysis for MACE are provided in Table 3. In the univariable analysis, significant predictors for MACE were age and regional MFR < 2. Multivariable analysis confirmed both as independent predictors.

**Table 3:** Univariable and multivariable cox-regression analysis for MACE

Predictor	Univariable				Multivariable			
	HR	95% CI	p-value	(i/m)q	HR	95% CI	p-value	(i/m)q
**Age (per year increase)**	**1.06**	**1.02–1.1**	**0.007**	**0.006**	**1.05**	**1.01–1.09**	**0.029**	**0.1**
Male gender	2.31	1.02–5.22	0.045	0.024	NA			
Body mass index (per kg/m^2^ increase)	1.03	0.98–1.09	0.232	0.065	NA			
Hypertension	2.03	0.87–4.78	0.103	0.053	NA			
Dyslipidemia	0.76	0.35–1.63	0.475	0.082	NA			
Diabetes	1.96	0.83–4.61	0.124	0.059	NA			
Positive family history	0.75	0.3–1.83	0.523	0.088	NA			
Smoking	1.17	0.53–2.58	0.703	0.094	NA			
Semiquantitative ischemia	0.97	0.29–3.22	0.967	0.100	NA			
Semiquantitative scar	2.28	0.97–5.37	0.059	0.035	NA			
LVEF (per 1% increase)^1^	0.97	0.93–1	0.044	0.018	NA			
Global MFR < 2	2.09	1.01–4.35	0.048	0.029	NA			
Global MFR (per 1 increase)	0.68	0.45–1.04	0.073	0.041	NA			
Global hMBF < 2 ml/min/g	1.96	0.93–4.12	0.075	0.047	NA			
Global hMBF (per 1 ml/min/g increase)	0.81	0.5–1.31	0.388	0.076	NA			
**Regional MFR < 2**	**4.17**	**1.44**–12**.08**	**0.009**	**0.012**	**3.44**	**1.17–10.11**	**0.024**	**0.05**
Regional hMBF < 2 ml/min/g	1.58	0.6–4.16	0.353	0.071	NA			

Variables significant at an FDR-controlled level q of 0.10 are highlighted in bold

^1^Available for 125 patients (83.3%)

*HR* hazard ratio, *CI* confidence interval, *NA* not applicable

Results of the cox-regression analysis for all-cause death are provided in Table 4. In the univariable analysis, significant predictors for death were age, semiquantitative scar, global MFR, global hMBF < 2 ml/min/g, and regional MFR < 2. Multivariable analysis confirmed age and regional MFR < 2 as independent predictors.

**Table 4:** Univariable and multivariable cox-regression analysis for all-cause death

Predictor	Univariable				Multivariable			
	HR	95% CI	p-value	(i/m)q	HR	95% CI	p-value	(i/m)q
**Age (per year increase)**	**1.08**	**1.03–1.14**	**0.003**	**0.006**	**1.07**	**1.01**–**1.12**	**0.015**	**0.05**
Male gender	2.13	0.83–5.50	0.118	0.059	NA			
Body mass index (per kg/m^2^ increase)	1.03	0.97–1.1	0.336	0.071	NA			
Hypertension	2.19	0.8–6.03	0.126	0.065	NA			
Dyslipidemia	0.88	0.36–2.26	0.878	0.088	NA			
Diabetes	2.58	0.99–6.73	0.052	0.041	NA			
Positive family history	0.94	0.34–2.56	0.899	0.094	NA			
Smoking	1.06	0.41–2.76	0.903	0.100	NA			
Semiquantitative ischemia	0.9	0.21–3.89	0.844	0.082	NA			
**Semiquantitative scar**	**3.06**	**1.16**–**7.98**	**0.022**	**0.029**	NS			
LVEF (per 1% increase)	0.96	0.92–1	0.053	0.047	NA			
Global MFR < 2	2.48	1.05–5.87	0.039	0.035	NA			
**Global MFR (per 1 increase)**	**0.48**	**0.27**–**0.83**	**0.009**	**0.012**	NS			
**Global hMBF < 2 ml/min/g**	**3.37**	**1.35**–**8.4**	**0.009**	**0.018**	NS			
Global hMBF (per 1 ml/min/g increase)	0.61	0.35–1.07	0.087	0.053	NA			
**Regional MFR < 2**	**6.24**	**1.45**–**26.9**	**0.014**	**0.024**	**4.72**	**1.07**–**20.7**	**0.040**	**0.1**
Regional hMBF < 2 ml/min/g	1.43	0.48–4.26	0.519	0.076	NA			

Variables significant at an FDR-controlled level q of 0.10 are highlighted in bold

*HR* hazard ratio, *CI* confidence interval, *NA* not applicable, *NS* non-significant

Multicollinearity between the quantitative PET metrics included in the regression analysis was found to be acceptable, although all parameters did correlate significantly (Supplementary Tables 3 and 4).

Of note, in a sub-analysis where early revascularizations (i.e., within 90 days after PET) were not excluded from the study population, semiquantitative ischemia (HR 2.35, 95% CI 1.14–4.85, p = 0.02) remained a significant predictor variable of MACE. The cardiac medication at the end of the follow-up of the population is displayed in Table 5.

**Table 5:** Cardiac medication at the end of follow-up

	All patients (n = 145)	Patients without MACE (n = 117)	Patients with MACE (n = 28)	p-value	χ2	(i/m)q
Antithrombotics	62 (42.8%)	46 (39.3%)	16 (57.1%)	0.087	2.9	0.038
Anticoagulants	42 (29%)	30 (25.6%)	12 (42.9%)	0.071	3.3	0.025
Betablockers	62 (42.8%)	48 (41%)	14 (50%)	0.389	0.7	0.075
Calcium antagonists	32 (22.1%)	24 (20.5%)	8 (28.6%)	0.356	0.9	0.063
ACE inhibitors	75 (51.7%)	61 (52.1%)	14 (50%)	0.839	< 0.1	0.100
Lipid-lowering drugs	73 (50.3%)	55 (47%)	18 (64%)	0.1	2.7	0.050
Nitrates	8 (5.5%)	6 (5.1%)	2 (7.1%)	0.675	0.2	0.088
**Diuretics**	**51 (35.2%)**	**34 (29.1%)**	**27 (60.7%)**	0.002	9.9	0.013

Values given are absolute numbers and (in parenthesis) percentages. Variables significant at an FDR-controlled level q of 0.10 are highlighted in bold. *ACE =* angiotensin-converting-enzyme. In five patients, it was not possible to obtain information about the medication at follow-up

## Discussion

The present study addresses the prognostic relevance of regional quantitative myocardial flow parameters assessed by ^13^N-ammonia PET-MPI in patients with suspected CAD. Our results demonstrate that regional MFR independently predicts MACE and all-cause death.

This finding extends the current knowledge on the value of global quantitative myocardial flow parameters such as global hMBF and MFR [7–11]. Many physiological factors, which affect microcirculation in particular (e.g., diabetes, hypertension, renal impairment) [22], affect myocardial perfusion globally. However, other factors are altering the coronary and, therefore, the myocardial blood flow on a regional level, such as plaques in the epicardial vessels or blood flow via collaterals [23, 24]. It may be hypothesized that slight changes in myocardial perfusion are only depictable through absolute myocardial flow quantification, rendering quantification more accurate than semiquantitative or qualitative analysis, which inarguably relies on a certain minimal threshold of relative perfusion differences. Consequently, and as demonstrated by our results, semiquantitative (i.e., visually perceivable) ischemia does not necessarily accompany slight perfusion restrictions as assessed quantitatively.

To our knowledge, only three studies have previously elaborated on the prognostic value of regional quantitative MBF parameters [25–27]:

Using ^82 ^Rb PET-MPI, Gould et al. reported that patients with at least one pixel with severely reduced coronary flow capacity (CFC) (i.e., MFR ≤ 1.27 and hMBF ≤ 0.83 ml/min/g) had a worse outcome compared to those with a normal CFC. Contrary to CFC, MFR is a widely used, easily applicable, and well-studied quantitative parameter derivable from the vast majority of presently available flow-analysis software solutions. Additionally, pixel-wise assessment of quantitative flow metrics is not available in all commonly used software solutions. However, and in general line with our findings, Gould et al. highlight the importance of regional MBF quantification and hint at its potential prognostic value.

Both Harjulahti et al. and Bom et al. previously found quantitative regional flow parameters to confer prognostic value for the prediction of myocardial infarction and death [26, 27]. However, several methodological differences must be noted between the present and both previous studies. In contrast to both studies, ^13^N-ammonia and not ^15^O-water was used as a PET tracer in the present study. Furthermore, in the Study by Bom et al., regional perfusion was defined as a continuous variable calculated as the average MFR values from the two adjacent segments with the lowest values within a vascular territory. In the present study, however, we have refrained from introducing regional perfusion as a continuous variable because our analysis revealed substantial multicollinearity, rendering the statistics potentially unreliable. Harjulahti et al. defined abnormal regional perfusion as a binary variable whereby a single segment with hMBF lower than 2.3 ml/g/min was considered as abnormal regional perfusion. By contrast, in the present study, a decrease in MFR or hMBF in at least two adjacent segments was required to be classified as reduced regional perfusion. It may be hypothesized that the methodology of our study may be less prone to subtle inhomogeneities and artifacts, but potentially less sensitive. Finally, contrary to Bom et al., the present study only included patients with suspected CAD. Additionally, it must be noted that, contrary to Harjulahti et al., our study population may comprise patients with non-obstructive CAD, which may also at least partly explain some differences between the two studies’ results. We feel that our naive population without interventions or known infarcts with possible effects on endothelial function or myocardial fibrosis allows for a more unbiased assessment of myocardial blood flow and its prognostic value. Accordingly, this exclusive patient selection may be regarded as a fundamental, intentionally chosen strength of our study.

Despite the methodological differences, the results from our study are essentially in line with the findings of Bom et al. and Harjulahti et al. regarding the prognostic value of regionally reduced myocardial perfusion. However, in the present study, only regionally but not globally reduced myocardial perfusion remained an independent predictor of future adverse events. By contrast, in the study of Harjulahti et al., both remained independent predictors, while Bom et al. did not report a regression analysis comparing regionally to globally reduced myocardial perfusion.

Semiquantitative ischemia or decreased global MFR was not associated with MACE in the present study, which stands in contrast to several previously published studies [8, 10, 28]. This may be convincingly explained by the fact that we excluded patients who underwent early revascularization from the analysis. Of note, semiquantitative scar, ischemia, and global MFR were reported to the referring physician. Hence, 12 patients with ischemia or decreased global MFR underwent early revascularization due to the reported imaging findings per se. By contrast, alterations in regional MFR were not reported and, therefore, did not trigger early revascularization or medical treatment in clinical routine. It can be hypothesized that such minor alterations in regional blood flow constitute very early changes that may confer future cardiovascular events if not treated. For example, a more aggressive prophylactic or therapeutic regimen may have been implemented in patients with semiquantitative ischemia and/or a decreased global MFR, both reported clinically, while any information on regional MFR (potentially constituting subtle ischemia) was not available and may not have prompted such measures. In fact, the present study’s retrospective nature may be perceived as a strength in that regional flow abnormalities were not reported to the treating physicians. Therefore, no potentially confounding prophylactic or therapeutic measures could have been initiated based on such findings.

The present study results are clinically relevant as they emphasize the importance of quantitative myocardial blood flow parameters compared to qualitative imaging findings, which are inherently limited by depending on a certain degree of relative perfusion differences. By contrast, PET-MPI with absolute flow quantification allows for the recognition of regionally limited and subtle pathological alterations in flow reserve, which may fall below the threshold needed for creating visually perceivable relative perfusion defects (i.e., semiquantitative ischemia) but may, nevertheless, identify patients who are at risk for future cardiovascular events with the potential to benefit from medical therapy very early along the ischemic cascade.

We acknowledge the following limitations: First, this study is a retrospective single-center study with all the inherent limitations of such a design. Second, we identified 180 patients from a registry spanning over a decade and containing approximately 1000 patients for the present study. The main reason for the relatively modest inclusion rate for the present study is that PET-MPI was not reimbursed in our country during 2005 and 2015. Hence, the vast majority included in the registry are patients with known severe CAD (e.g., with a history of coronary artery bypass grafting), not fitting the inclusion criteria of this study. Additionally, it may be perceived as a limitation that established thresholds for MBF and MFR were applied, as previously documented [8] and as recommended by the ASNC [7]. We intentionally refrained from calculating population-specific thresholds for this study for hMBF and MFR for the prediction of MACE and death so as to preserve the comparability of our results.

In conclusion, this study demonstrates that in patients with suspected CAD, a decreased regional MFR as assessed by ^13^N-ammonia PET-MPI confers prognostic value by identifying patients at increased risk for future adverse cardiac outcomes and all-cause death. Our results underline the findings from previous studies and hint at a potential clinical benefit that may be derived from regional quantitative blood flow assessment as an adjunct to global quantitative and qualitative analysis of PET-MPI.

## Supplementary Information

Below is the link to the electronic supplementary material.

Supplementary file1 (DOCX 17 KB)

## Data Availability

The datasets used and/or analyzed during the current study are available from the corresponding author on reasonable request.
