# Supplementary material for: Prognostic value of regional myocardial flow reserve derived from 13N-ammonia positron emission tomography in patients with suspected coronary artery disease
Source: Eur J Nucl Med Mol Imaging. 2021 Jun 30;49(1):311–20. doi: 10.1007/s00259-021-05459-0 (PMC8712296; doi:10.1007/s00259-021-05459-0)
Supplement: Supplementary file 1 — Supplementary file1 (DOCX 17 KB) [file 259_2021_5459_MOESM1_ESM.docx]

**Supplementary Table 1 . Multivariable regression analysis models for death**

| **Predictor** | **HR** | **95% CI** | **p-value** | **Chi-Square (sig)** | **∆Chi-Square (sig)** |
| --- | --- | --- | --- | --- | --- |
| Model 1 | | | | 18.26 (p=0.003) | 18.26 (p=0.003) |
| Age | 1.06 | 1.01-1.11 | 0.028 |  |  |
| Semiquantitative scar | 1.36 | 0.49-3.76 | 0.551 |  |  |
| Global MFR (per 1 increase) | 0.86 | 0.36-1.94 | 0.721 |  |  |
| Global hMBF < 2 ml/min/g | 1.6 | 0.54-4.73 | 0.393 |  |  |
| Regional MFR < 2 | 3.08 | 0.51-18.72 | 0.223 |  |  |
| Model 2 | | | | 18.14 (p=0.001) | 0.12 (p=0.719) |
| Age | 1.06 | 1.01-1.11 | 0.029 |  |  |
| Semiquantitative scar | 1.39 | 0.51-3.81 | 0.517 |  |  |
| Global hMBF < 2 ml/min/g | 1.71 | 0.61-4.75 | 0.308 |  |  |
| Regional MFR < 2 | 3.8 | 0.79-18.34 | 0.096 |  |  |
| Model 3 | | | | 17.73 (p=0.001) | 0.41 (p=0.524) |
| Age | 1.06 | 1.01-1.12 | 0.021 |  |  |
| Global hMBF < 2 ml/min/g | 1.85 | 0.69-4.96 | 0.222 |  |  |
| Regional MFR < 2 | 3.48 | 0.72-16.71 | 0.119 |  |  |
| Model 4 | | | | 16.15 (p<0.001) | 1.58 (p=0.208) |
| Age | 1.07 | 1.01-1.12 | 0.015 |  |  |
| Regional MFR < 2 | 4.72 | 1.07-20.7 | 0.040 |  |  |

A stepwise backward conditional approach was applied. Significant predictor variables in univariable analysis were included in model 1. In each subsequent model, the non-significant variable with the highest p-value was excluded. Overall model fit compared to the null model is given by chi-square values. ∆Chi-Square: Changes in the Chi-square values compared to the previous model. Sig: Significance.

**Supplementary Table 2. Events and incidence rates stratified by groups**

|  | **All Patients (n=150)** | **MFR Group 1 (n=52)** | **MFR Group 2 (n=49)** | **MFR Group 3 (n=49)** | **hMBF Group 1 (n=34)** | **hMBF Group 2 (n=48)** | **hMBF Group 3 (n=68)** |
| --- | --- | --- | --- | --- | --- | --- | --- |
| **MACE, n (%)** | 30 (20.0) | 5 (9.6) | 11 (22.4) | 14 (28.6) | 5 (14.7) | 9 (18.8) | 16 (23.5) |
| **Annual MACE rate, %** | 3.7 | 1.6 | 4.7 | 5.6 | 2.5 | 3.1 | 5.2 |
| **Death, n (%)** | 21 (14.0) | 2 (3.8) | 8 (16.3) | 11 (22.4) | 4 (11.8) | 3 (6.3) | 14 (20.6) |
| **Annual death rate, %** | 2.5 | 0.6 | 3.2 | 4.1 | 1.9 | 1.0 | 4.4 |

**Supplementary Table 3. Correlation of quantitative PET metrics**

|  | **Global MFR** | **global MFR < 2** | **Regional MFR < 2** | **Global hMBF** | **Global hMBF < 2 ml/min/g** | **Regional hMBF <2 ml/min/g** |
| --- | --- | --- | --- | --- | --- | --- |
| **Global MFR** |  | -0.667 (p<0.001) | -0.616 (p<0.001) | 0.471 (p<0.001) | -0.432 (p<0.001) | -0.290 (p<0.001) |
| **global MFR < 2** | -0.667 (p<0.001) |  | 0.507 (p<0.001) | -0.363 (p<0.001) | 0.394 (p<0.001) | 0.241 (p=0.003) |
| **Regional MFR < 2** | -0.616 (p<0.001) | 0.507 (p<0.001) |  | -0.441 (p<0.001) | 0.466 (p<0.001) | 0.375 (p<0.001) |
| **Global hMBF** | 0.471 (p<0.001) | -0.363 (p<0.001) | -0.441 (p<0.001) |  | -0.708 (p<0.001) | -0.546 (p<0.001) |
| **Global hMBF < 2 ml/min/g** | -0.432 (p<0.001) | 0.394 (p<0.001) | 0.466 (p<0.001) | -0.708 (p<0.001) |  | 0.493 (p<0.001) |
| **Regional hMBF < 2 ml/min/g** | -0.290 (p<0.001) | 0.241 (p=0.003) | 0.375 (p<0.001) | -0.546 (p<0.001) | 0.493 (p<0.001) |  |

The given values are correlation coefficients and, in parenthesis, p-values. Variables were tested for significant correlation using the kendall-tau test.

**Supplementary Table 4. Multicollinearity analysis of quantitative PET metrics for MACE and Death**

| **Variable** | **VIF for MACE** | **VIF for Death** |
| --- | --- | --- |
| **Global MFR** | 3.6 | 3.6 |
| **Global MFR < 2** | 1.9 | 1.9 |
| **Regional MFR < 2** | 2.1 | 2.1 |
| **Global hMBF** | 4.6 | 4.6 |
| **Global hMBF < 2 ml/min/g** | 2.8 | 2.8 |
| **Regional hMBF < 2 ml/min/g** | 1.9 | 1.9 |

The given values are variation inflation factors (VIF). All listed variables were included in the model, while the dependent variable was MACE-free and overall survival.
